# In Silico Identification of Novel Derivatives of Rifampicin Targeting Ribonuclease VapC2 of *M. tuberculosis* H37Rv: Rifampicin Derivatives Target VapC2 of *Mtb* H37Rv

**DOI:** 10.3390/molecules28041652

**Published:** 2023-02-08

**Authors:** Satyamvada Maurya, Amita Jain, Md Tabish Rehman, Ali Hakamy, Farkad Bantun, Mohamed F. AlAjmi, Vineeta Singh, Aafreen Zehra, Feroz Khan, Shafiul Haque, Bhartendu Nath Mishra

**Affiliations:** 1 Department of Biotechnology, Institute of Engineering & Technology, Dr. A.P.J. Abdul Kalam Technical University, Lucknow 226021, India; 2 Department of Microbiology, King George Medical University, Lucknow 226003, India; 3 Department of Pharmacognosy, College of Pharmacy, King Saud University, Riyadh 11451, Saudi Arabia; 4 Respiratory Therapy Department, Faculty of Applied Medical Sciences, Jazan University, Jazan 45142, Saudi Arabia; 5 Department of Microbiology, Faculty of Medicine, Umm Al-Qura University, Makkah 21955, Saudi Arabia; 6 Technology Dissemination and Computational Biology Unit, CSIR–Central Institute of Medicinal and Aromatic Plants, Lucknow 226015, India; 7 Research & Scientific Studies Unit, College of Nursing & Allied Health Sciences, Jazan University, Jazan 45142, Saudi Arabia; 8 Gilbert and Rose-Marie Chagoury School of Medicine, Lebanese American University, Beirut 1102 2801, Lebanon; 9 Centre of Medical and Bio-Allied Health Sciences Research, Ajman University, Ajman 13306, United Arab Emirates

**Keywords:** molecular docking, tuberculosis, *Mycobacterium tuberculosis*, root mean square deviation, radius of gyration, root mean square fluctuation, ADMET

## Abstract

The emergence of multi-drug-resistant *Mycobacterium tuberculosis* (*Mtb*) strains has rendered many of the currently available anti-TB drugs ineffective. Hence, there is a pressing need to discover new potential drug targets/candidates. In this study, attempts have been made to identify novel inhibitors of the ribonuclease VapC2 of *Mtb* H37Rv using various computational techniques. Ribonuclease VapC2 *Mtb* H37Rv’s protein structure was retrieved from the PDB databank, 22 currently used anti-TB drugs were retrieved from the PubChem database, and protein–ligand interactions were analyzed by docking studies. Out of the 22 drugs, rifampicin (RIF), being a first-line drug, showed the best binding energy (−8.8 Kcal/mol) with *Mtb* H37Rv VapC2; hence, it was selected as a parent molecule for the design of its derivatives. Based on shape score and radial plot criteria, out of 500 derivatives designed through SPARK (Cresset^®^, Royston, UK) program, the 10 best RIF derivatives were selected for further studies. All the selected derivatives followed the ADME criteria concerning drug-likeness. The docking of ribonuclease VapC2 with RIF derivatives revealed the best binding energy of −8.1 Kcal/mol with derivative 1 (i.e., RIF-155841). A quantitative structure–activity relationship study revealed that derivative 1’s activity assists in the inhibition of ribonuclease VapC2. The stability of the VapC2–RIF155841 complex was evaluated using molecular dynamics simulations for 50 ns and the complex was found to be stable after 10 nsec. Further, a chemical synthesis scheme was designed for the newly identified RIF derivative (RIF-155841), which verified that its chemical synthesis is possible for future in vitro/in vivo experimental validation. Overall, this study evaluated the potential of the newly designed RIF derivatives with respect to the *Mtb* VapC2 protein, which is predicted to be involved in some indispensable processes of the related pathogen. Future experimental studies regarding RIF-155841, including the exploration of the remaining RIF derivatives, are warranted to verify our current findings.

## 1. Introduction

Tuberculosis (TB) is still a serious public health concern and is one of the most prevalent infectious diseases, causing mortality worldwide. According to estimates, in 2020, about 10 million people fell ill with TB worldwide and 1.5 million died from the disease. It is the thirteenth leading cause of death and the second leading infectious disease after COVID-19 (https://www.who.int/news-room/fact-sheets/detail/tuberculosis (accessed on 27 October 2022)). TB incidence may rise globally in 2022 and 2023 as a result of the COVID-19 pandemic [1,2]. A variety of therapeutic options are available for the treatment of TB, including first-line medicines such as isoniazid (INH), rifampin (RIF), pyrazinamide (PZA), ethambutol (EMB) and streptomycin (SM); however, this drug regimen often fails for various reasons. The emergence of multidrug-resistant TB (MDR-TB) due to relapse and the spread of the version of the disease resistant to (at least) isoniazid (INH) and rifampicin (RIF) are causes for major concern, as these factors necessitate second-line medications that are difficult to procure and are relatively more toxic and costly than the first-line drugs. The second-line anti-TB medicines can be sub-divided into the following groups: (i) fluoroquinolones, e.g., ofloxacin (OFX), levofloxacin (LEV), moxifloxacin (MOX), and ciprofloxacin (CIP), and (ii) injectable aminoglycosides, e.g., kanamycin (KAN), amikacin (AMK), and capreomycin (CAP). In addition, (iii) less-effective second-line medicines include ethionamide (ETH)/prothionamide (PTH), cycloserine (CS)/terizidone, and p-aminosalicylic acid (PAS). Hence, the diagnosis as well as treatment of drug-susceptible/single-drug-resistant TB is vital to avert the emergence of MDR-TB. Additionally, there have been several occurrences of extensively drug-resistant TB (XDR-TB), wherein *Mtb* is resistant to either isoniazid or rifampicin (similar to MDR-TB), any fluoroquinolone, and at least one of the three second-line anti-TB drugs.

Drug-resistance against TB therapy can be instigated by a variety of conditions, one of which being a patient’s inability to adhere to the treatment protocols and schedules, resulting in *Mtb* isolates that are resistant to currently approved drugs [3]. As a result, novel strategies for combating TB are required, thereby necessitating a reassessment of the medical treatment methods and warranting the development of more potent anti-TB drugs. Thus, the contrivance of a strategy for developing novel antimicrobials by identifying and investigating novel biological targets is crucial to combat the pathogen. The same can also be attained by evaluating the existing drugs or their derivatives against the newly identified bio-targets [4]. Rifampicin (RIF) is one such established anti-TB drug that has shown superb sterilizing activity and acts by binding with RNA polymerase β-subunit (*rpoB*), which leads to the inhibition of the transcription process, thereby killing the pathogen. RIF exclusively works against actively growing and slowly metabolizing (non-growing) bacilli and causes comparatively fewer adverse reactions.

Recently, the proteins involved in bacterial cytokinesis have been investigated with regard to their role as potential targets, as these are necessary for bacterial multiplication and growth. Ribonuclease VapC2 is one such crucial protein. The VapC2 protein is a toxic component of the type II toxin–antitoxin (TA) system and acts as RNase. The expression of VapC2 in *M. smegmatis* inhibits translation, growth, and colony formation [5]. The VapC2 (Rv0301) protein has 141 amino acids. Small protein domains, such as PIN (PilT N-terminus), with strictly conserved acidic residues are also found in vapC2 toxin and form part of the toxin–antitoxin (TA) operon [6]. This domain of *Mtb* Rv0300 is similar to Rv2757c, Rv0229c, Rv2546, and others. This PIN domain area is considered to be a possible *Mtb*-complex-specific genomic island [7]. The significance of VapC2 and other cytokinesis proteins in bacterial survival has made them promising novel targets for antibiotic research [8].

Considering the aforementioned facts regarding the anti-TB potential of RIF and the indispensable role of Ribonuclease VapC2 for the survival of the pathogen, this study was conducted with the aim of designing novel RIF derivatives, assessing protein (Ribonuclease VapC2)–ligand (RIF derivatives) interactions, and evaluating their bioavailability using computational approaches to molecular docking and molecular dynamics (MD) simulations supported with ADMET analysis.

## 2. Results and Discussion

### 2.1. Ribonuclease VapC2 Protein

**Figure 1:**
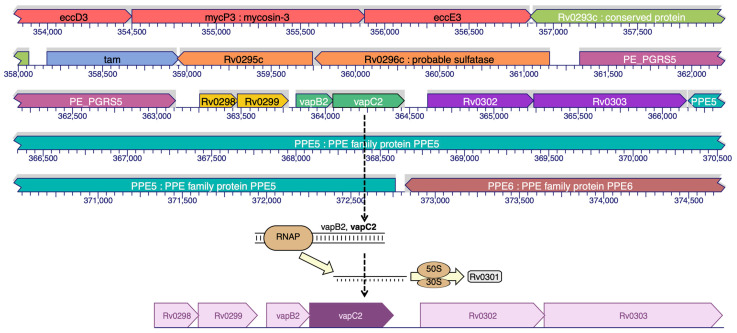
Binding site prediction of VapC2 protein.

Ribonuclease VapC2 acts as RNase. The TA (toxin–antitoxin) system in *Mtb* can contribute to its pathogenesis and also aids in the translation of *Mtb* [9]. Figure 1 shows the location of the VapC2 protein. The VapC2 protein has an area (SA) of 1089.651 and a volume (SA) of 2885.670.

### 2.2. Ligand Growing Experiment

**Figure 2:**
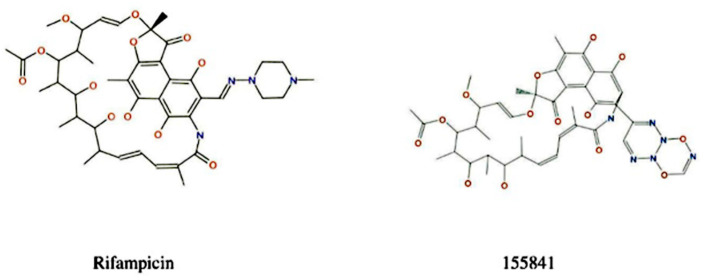
Reference compound and its most active derivative.

During the selection of a lead molecule, 22 currently used drugs were docked against VapC2 protein and RIF was found to possess the best binding energy, namely, −8.8 kcal/mol (Supplementary Materials: Table S1). Although the binding energies of Bedaquiline (−8.7 kcal/mol) and Amikacin (−8.7 kcal/mol) were almost the same as RIF, we proceeded with RIF as it belongs to the first-line anti-TB drug family. The selected RIF drug was used for the design of inhibitors (RIF-derivatives) against *Mtb* using SPARK 10.5.6 (Figure 2); as a result, a library of 500 novel RIF derivatives was created. Following the filtration criteria of the shape score, field score, radial-plot, and Lipinski’s “rule of five”, a total of 10 RIF compounds (derivatives) were selected for further analysis. The derivative RIF-155841 was found to be the most active. The structural details of the top 10 selected RIF derivatives (2–10) are provided in Supplementary Materials: Figure S1.

### 2.3. ADME Properties

The bioavailability of the selected RIF derivatives was assessed according to their size, lipophilicity, solubility, polarity, flexibility, and saturation (Table 1).

**Figure 3:**
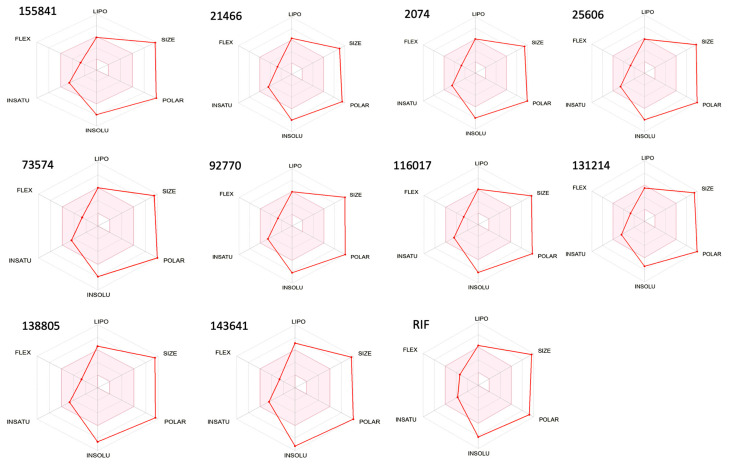
Bioavailability radar of RIF and its ten selected derivatives. Note: The pink area represents the optimal range for each of the properties.

The bioavailability radar of RIF and its selected derivatives has been depicted in Figure 3. According to their ADME characteristics, the results for all 10 RIF compounds were quite favorable [10] and suggest that these derivatives can be orally bioavailable, i.e., they will enter the systemic circulation in a tolerable amount when taken orally. RIF and its derivatives displayed almost similar ADME properties (Table 1).

### 2.4. Prediction of Antimicrobial Activity of RIF Derivatives Using QSAR Modeling

The developed QSAR model showed an accuracy of 73.8% (regression coefficient R^2^ = 0.738; LOO cross validation regression coefficient R^2^_cv_ = 0.65). A total of nine molecular descriptors, viz., GATS6s, MDEC-34, AATS6p, SpMax2_Bhv, AATS8i, maxHsOH, minHBint8, VR2_Dzs, and MATS8i, were found to be significantly associated with antimicrobial activity. The model equation given below shows the relationship between the experimental in vitro activity [IC_50_ nM] and the chemical descriptors, which were dependent and independent variables, respectively.

Predicted Activity Log10 (IC_50_) = (−1.835869322 × GATS6s) + (0.46431287 × MDEC-34) + (3.368747089 × AATS6p) + (−5.792245074 × SpMax2_Bhv) + (0.024503123 × AATS8i) + (−0.980285851 × maxHsOH) + (−0.143211 × minHBint8) + (−0.011437386 * VR2_Dzs) + (1.861532898 × MATS8i) + 18.4871726

**Figure 4:**
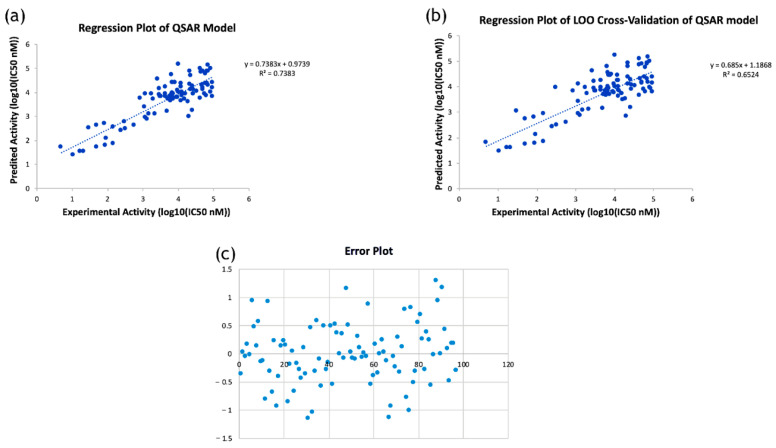
(**a**) Regression plot of the developed QSAR model; (**b**) Regression plot of leave-one-out cross-validation of the developed QSAR model; (**c**) Error plot showing the difference between predicted activity values and experimental activity values.

Here, R^2^ is 0.74, which indicates that the correlation between the activity (dependent variable) and the descriptors (independent variables) for the training data set compounds was 74% (Figure 4a), and the R^2^_LOOCV_ is 0.65 (Figure 4b). The difference between the predicted activity values and the experimental activity values is shown in Figure 4c. From the above equation, it is apparent that the molecular descriptors GATS6s, SpMax2_Bhv, maxHsOH, minHBint8, and VR2_Dzs are negatively correlated with the antimicrobial activity, i.e., an increase in the values of these descriptors will decrease this activity. Whereas the descriptors MDEC-34, AATS6p, AATS8i and MATS8i are positively correlated with antimicrobial activity, i.e., an increase in the values of these descriptors will increase such activity. Finally, the developed QSAR model was used to predict the toxic potential of the newly designed RIF derivatives against *Mtb* H37Rv (Table 2).

### 2.5. Molecular Docking

The binding site of the VapC2 protein was docked with the 10 selected RIF analogues. Derivatives 1 and 2 had the best docking values (−8.1 and −7.9) out of the ten selected compounds (Table 3).

**Figure 5:**
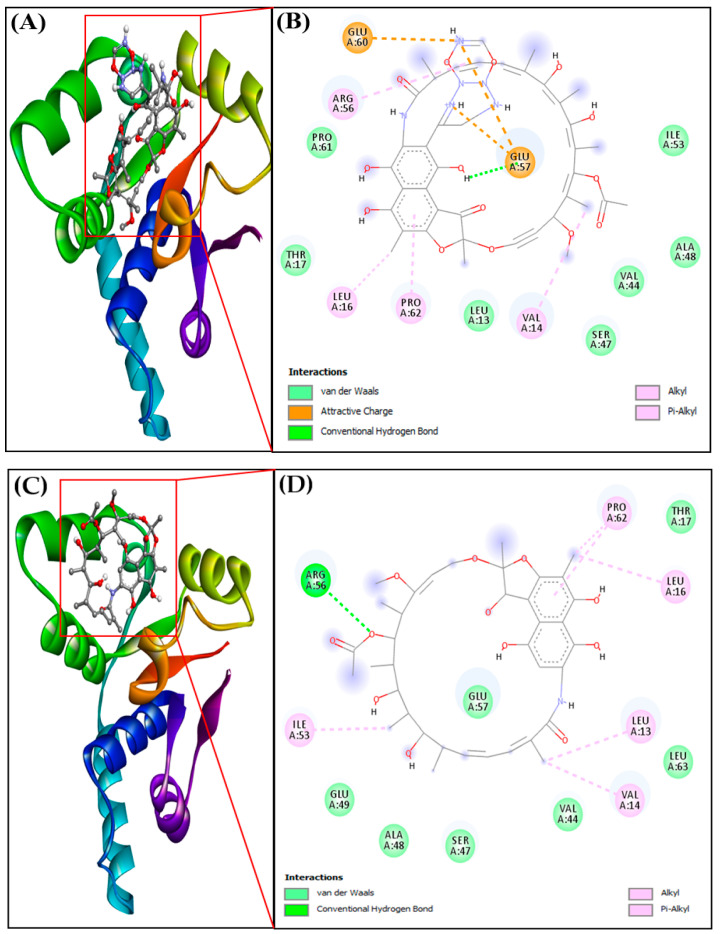
(**A**) Docked complex of protein-ligand RIF155841-3H87, (**B**) 2D interaction of protein-ligand RIF155841-3H87, (**C**) Docked complexes of protein-ligand RIF-3H87 (VapC2), (**D**) 2D interaction of protein-ligand RIF-3H87 (VapC2).

Derivatives 3–6 had nearly the same docking energy of about −7.8 kcal/mol, which suggested that the nature of their side chains were nearly the same; hence, no major change was observed in terms of binding affinity. However, Derivatives 7–10 had reduced binding affinity towards the VapC2 protein, with docking energy values of −7.7, −7.6, −7.2, −6.9, and −6.7 kcal/mol. RIF had a docking energy value of −6.8 kcal/mol, which indicated a lower binding affinity in comparison with its derivatives. The docking of RIF and its derivative 1 with 3H87 protein is shown in Figure 5A–D, respectively. RIF155841 (derivative 1) pose 1 showed the best binding ability (−8.1 kcal/mol) among all the other poses (Supplementary Materials: Table S3).

### 2.6. Protein–Ligand Intermolecular Interaction Analysis

The importance of hydrogen bonding in the docked complex (protein–ligand interactions) was explored further (Table 4). RIF155841-3H87 (VapC2) showed a binding affinity (M^−1^) of 8.73 × 10^5^, and this value was 9.72 × 10^4^ for the RIF-3H87 (VapC2) complex (Table 4). The corresponding ligand–protein interactions are generally characterized by typical hydrogen bonds, alkyl bonds, and attracting charges. The amino acids leucine, valine, proline, and arginine were involved in the alkyl interactions with the RIF derivative, whereas glutamic acid was involved in charge interactions. The introduction of a ring structure in the side-chain increased the number of charge interactions, which, in turn, stabilized the complex and led to better binding affinity.

### 2.7. Molecular Dynamics Analysis

**Figure 6:**
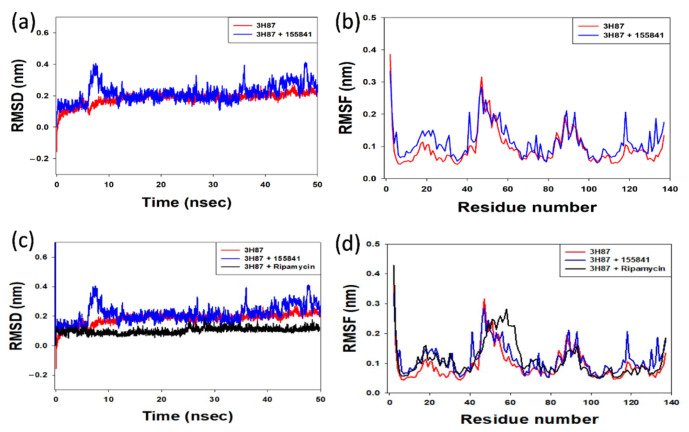
(**a**) Comparative RMSD plot of the protein–ligand (VapC2-RIF 155841) complex; (**b**) RMSF plot of alpha carbon atoms against the residue number of VapC2 bound to the ligand RIF 155841 (**c**) Comparative RMSD plot of the protein–RIF (VapC2-RIF) complex; (**d**) RMSF plot of alpha carbon atoms against the residue number of VapC2 bound to RIF.

The Root-Mean-Square Deviation (RMSD) analysis of the protein–ligand complexes was computed for 50 nsec. The VapC2-RIF 155841 complex converged and reached stability at around 12 ns, as illustrated in Figure 6a, and remained stable until the end of the simulation experiment. For a better understanding of the stability of the docked complexes, the RMSD was determined for the RIF 155841-3h87 (VapC2) complex. The RIF 155841-3h87 (VapC2) complex had an average RMSD value of 0.4. The alpha carbon atoms of the Vapc2-155841 complex residues are shown in Figure 6b as a root-mean-square fluctuation (RMSF) plot. The residues 0–20, 40–60, and 85–95 showed distinct variations in terms of the RIF 155841 compound. A significant constraint was identified, as these areas contain residues at the binding locations. The RIF-3h87 (VapC2) docked complex was also stable at 12 ns and its RMSD value was 0.2 (Figure 6c,d).

### 2.8. Chemical Synthesis of Potentially Identified Newly Designed RIF Derivative

**Figure 7:**
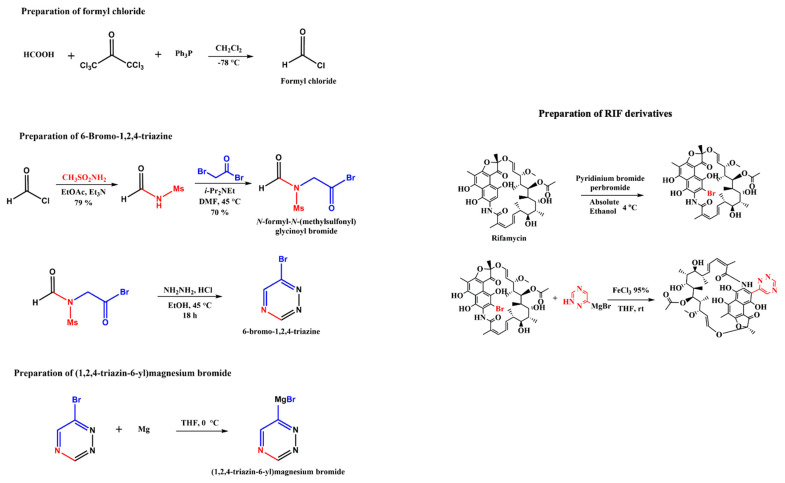
Chemical synthesis scheme for RIF 155841 derivative.

Further, the design of a successful chemical synthesis scheme for the identified RIF 155841 derivative against VapC2 verified the practicability of the development of this potential anti-TB drug (Figure 7). The proposed chemical synthesis scheme can be further implemented for compound synthesis followed by experimental validation studies. Further in-silico NMR (https://www.nmrdb.org (accessed on 29 October 2022)) and MS characterization of the RIF 155841 derivative were performed, and pertinent data have been provided in Supplementary Materials: Figure S2 and Figure S3, respectively.

## 3. Materials and Methods

### 3.1. Protein Retrieval

Ribonuclease VapC2 (UniProt id: P9WFB9) protein was retrieved from Protein Data Bank (PDB) (https://www.rcsb.org/ (accessed on 23 September 2019), which is available under the following identification number: PDB ID 3H87 (Rv0301-Rv0300, Toxin–antitoxin complex from *Mtb*). Rv0301-Rv0300, an *Mtb* VapBC TA complex with 1.49-resolution crystal structure, offers three major functions: anti-toxin inhibition, RNase activity, and promoter DNA binding. *VapC2* gene is a 426 bp nucleotide found in the cytosol and plays a variety of roles in pathogen virulence, detoxification, and adaptation.

### 3.2. Prediction of the Binding Site

CASTp 3.0 was used to predict VapC2′s binding pocket [11]. The pocket with the greatest area and volume was considered to be the most likely binding pocket of the Ribonuclease VapC2 protein of *Mtb*.

### 3.3. Ligand Selection

The 3D structures of 22 known (currently in use) anti-TB drugs (first-line, second-line, oral, and injectable) were retrieved from the PubChem database (https://pubchem.ncbi.nlm.nih.gov (accessed on 10 October 2019)) and blind docking was performed against VapC2 (3H87) protein (Supplementary Materials: Table S1). To design and synthesize a potential derivative(s) against VapC2 (3H87) protein, the best-docked compound (drug) was chosen.

### 3.4. Derivative Designing

SPARK (Cresset^®^, Royston, UK) program was utilized to design novel anti-TB drug derivatives of RIF [12,13]. To promote ligand design, rifampicin (RIF) was employed as a reference molecule. Before use, the starting and reference molecules were three-dimensionally aligned. SPARK was used in conjunction with three databases: ChEMBL common with 58,924 fragments and Commercial Very Common and Common with 20,561 and 42,778 fragments, respectively. Both the distances and angles were considered when evaluating the matches. The electrostatic status and similarities of these newly designed RIF derivatives were compared with the starting structure.

### 3.5. In Silico Prediction of Drug Likeness, Bioavailability and Toxicity

The SwissADME (http://www.swissadme.ch (accessed on 16 December 2019)) web application was used to appraise the ADME (absorption, distribution, metabolism, and excretion) pharmacokinetic properties of the newly designed RIF-compounds [13]. The SwissADME uses canonical SMILES (simplified molecular input line entry specification) together with the Bioavailability Radar to describe a chemical molecule. The OpenBabel API (version 2.3.0, 2012) was used to compute all descriptors and molecular parameters. A linear skin permeation technique based on Potts and Guy’s quantitative structure–property relationship (QSPR) model, which links the decimal logarithm of Kp to MW and logP, was presented in the pharmacokinetics section. To predict Kp, multiple linear regression was employed [14]. For drug-likeness pre-screening investigations, Lipinski’s “rule of five” (Pfizer) [15,16] and Ghose’s [17], Veber’s [18], Egan’s [19], and Muegge’s [20] guidelines were followed. The admetSAR (version 2.0) http://lmmd.ecust.edu.cn/admetsar2.html (accessed on 11 January 2021) online tool and ADMETLab were used to estimate the toxicological profiles of the designed RIF derivatives [21,22].

### 3.6. Quantitative Structure–Activity Relationship (QSAR) Analysis

*Data collection:* Antimicrobial structure-versus-activity data for *Mtb* H37Rv were downloaded from ChEMBL database (https://www.ebi.ac.uk/chembl/ (accessed on 18 June 22)). The retrieved data set was filtered by removing the outlier and redundant data; at the end, a total of 97 compounds were considered for the training set. Bioactivity was expressed in the form of IC_50_ (nM) values. The data were normalized by converting the values into log_10_ (base 10) form.

*Structure drawing and cleaning:* Drawing and geometry cleaning of compounds with antimicrobial activity were performed using ChemDraw Professional version 15.0 software (PerkinElmer Informatics, Waltham, MA, USA). The two-dimensional (2D) structures were transformed into three-dimensional (3D) structures using Chem3D version 15.0. The 3D structures were then subjected to energy minimization using molecular mechanics-2 (MM2).

*Descriptor calculation and model development:* Molecular descriptors were calculated using PaDEL-Descriptor program (http://www.yapcwsoft.com/dd/padeldescriptor/ (accessed on 28 August 2022)). The descriptors were filtered through the observation of an inter-descriptor correlation matrix. The QSAR model’s generation was based on step-wise multiple linear regression method. A comparison of experimental and predicted activities of the training data set based on the QSAR model is shown in Supplementary Materials: Table S2.

*Cross-validation of the model:* The developed model was cross-validated by Leave-one-out cross-validation (LOOCV) method. For the data set N, (N − 1) was considered as a ‘training set’ and the remaining one was designated as a ‘test set’. Hence, the training and testing phases were repeated N times, and in this manner, all the data passed through the testing process [23].

### 3.7. Molecular Docking

For molecular docking experiments, AutoDock tool was used. Molecular docking includes phases such as protein preparation, ligand preparation, grid generation, and molecular docking/interaction analysis. A grid box was created using Autodock version 4.2.6. The grid size was set to 60 × 60 × 60 for points x, y, and z, respectively, with a grid spacing of 0.375, and the grid center was set at −1.095, −1.554, and 3.894 for x, y, and z dimensions, respectively. To reduce computing time, a score grid was generated using the ligand structure. The information on the proteins and ligands, as well as grid box attributes in the configuration file, were used for docking studies performed through AutoDock/Vina version 4.2.6. Heuristic local search global optimizer was utilized in AutoDock/Vina version 4.2.6. During the docking process, both, the proteins and the ligands were considered ‘rigid’ entities. The data with a positional root-mean-square deviation (RMSD) of less than 1.0 were grouped and represented by the binding free energy with the lowest value. The position with the lowest binding energy or affinity was extracted and aligned with the receptor structure for the future analysis. The ligand pose with the best binding affinity was extracted from the docked complex [24,25] for further studies.

### 3.8. Molecular Dynamics Simulation

Molecular dynamics (MD) simulations were performed using GROMACS (Version 5.1.3). Previously retrieved docked complexes were used as ‘input’ for MD simulations. Protein topology was generated using gromos43a1.ff force field via pdb2gmx module. Following the introduction of the protein–ligand complex, a unit cell was filled with water. Afterwards, Na^+^ was added to neutralize the system, and the steepest descent energy method was adopted to minimize the system. GROMACS calculates electrostatic and Van der Waals interactions as well as Particle Mesh Ewald (PME) energy. All complexes were equilibrated for 100 psec under constant volume (NVT) and constant pressure (NPT) conditions with position restraints and applied to the ligands at the same time [26]. Later, the system was subjected to 50 nsec MD production run. The ligand RMSD, C-alpha backbone, and RMSF graphs were created using XMGRACE version 5.1.25.

### 3.9. Chemical Synthesis of RIF Derivatives

The best-docked RIF derivative was subjected to design of chemical synthesis process. The possible chemical synthesis procedure of RIF derivatives is shown in the chemical scheme provided in Figure 7. The side chain that reacts with RIF can be prepared in a three-step process. In the first step, formyl chloride can be synthesized using formic acid and hexachloro acetone. This formyl chloride can undergo multiple reactions and forms 6-Bromo-1,2,4-triazine. This 6-Bromo-1,2,4-triazine further reacts with Magnesium under the reaction condition of diethyl ether or tetrahydrofuran P(THF) and 0 °C to yield Grignard reagent. RIF primarily transforms into its Bromo derivative to react further. Bromo-RIF reacts with Grignard reagent under the reaction condition of FeCl_3_-THF at room temperature and thus yields the final RIF derivative. Further, in silico chemical characterization of RIF derivative was performed to ascertain its chemical properties.

## 4. Conclusions

Understanding the interactions between proteins and lead molecules is vital to the development of more targeted and potent inhibitors. The major goal of this study was to find potential inhibitor(s) of the *Mtb* VapC2 protein using newly designed RIF derivatives, followed by molecular docking and MD simulations. This study provides a remarkable insight into what to expect when these inhibitors interact with the VapC2 protein. Out of the ten selected RIF derivatives, only one derivative (RIF 155841) scheme was successfully prepared for chemical synthesis. The newly designed RIF derivative’s ability to inhibit *Mtb* H37Rv was predicted by the constructed QSAR model. Molecular docking analysis showed that all the selected RIF derivatives were found to be active with respect to binding with the VapC2 protein and showed quite a strong affinity for VapC2. The precise binding at the active site of VapC2 protein with RIF derivative 2 and RIF derivative 3 constituted the greatest binding affinities while RIF derivative 7 bore the lowest such value. All the RIF derivatives had decent binding energy, especially VapC2-155841, which had the highest binding energy and ligand efficiency. Bioavailability analysis revealed that all of the RIF derivatives examined were potentially suitable for oral administration and must be further explored. In addition, the MD analysis revealed that the VapC2-RIF 155841 complex was quite stable at approximately 12 nsec. The RMSD values indicated that the binding of the VapC2-RIF155841 complex had been stabilized without any conformational shift. Overall, the newly designed RIF derivatives altered VapC2′s structural conformation and showed good outcomes as a prospective anti-TB drug candidate, while derivative RIF 155841 produced the best results. This computational investigation reveals potential new drug candidates and their associated drug–target intermolecular interactions, further paving the way for the experimental validation of the current findings.

## Figures and Tables

**Table 1:** Pharmacochemical properties of the selected ligands (RIF derivatives).

Parameters	Optimum Range	Rifampicin	RIF 155841	RIF 2074	RIF 21466	RIF 25606	RIF 73574	RIF 92770	RIF 116017	RIF 131214	RIF 138805	RIF 143641
Size	MW between 150 and 500 g/mol	822.94	836.84	833.83	764.81	804.84	832.85	804.84	848.91	837.89	804.84	803.85
Number of Rotatable bonds	Not more than 9 rotatable bonds	5	4	4	4	4	4	4	4	4	4	4
#H-bond acceptors	No more than 10 hydrogen bond acceptors	14	17	17	14	15	16	15	15	15	15	14
#H-bond donor	No more than 5 hydrogen bond donors	6	6	6	6	7	6	6	6	7	7	6
XLOGP3	Between −0.7 and +5.0	5.46	7.34	4.71	5.53	5.1	4.72	4.96	4.94	5.1	4.16	6.03
ESOL Log S	Less than 6	−8.18	−9.64	−7.95	−8	−8.01	−7.95	−7.92	−8.19	−8.21	−7.42	−8.59
TPSA (Å2)	Must be between 20 and 130	220.15	275.08	270.44	227.34	256.02	261.71	244.64	276.81	288.44	260.18	227.59
Fraction of Csp3	Must not be less than 0.25	0.53	0.45	0.43	0.45	0.44	0.43	0.44	0.43	0.44	0.45	0.42

**Table 2:** Predicted activity of newly designed, selected RIF derivatives.

Name/Structure of RIF Derivative	Predicted Activity Log10 (IC_50_)	Predicted Activity (IC_50_ nM)
RIF 2074	4.42	26,376.59
RIF 21466	4.20	15,997.57
RIF 25606	3.60	3983.70
RIF 73574	4.00	10,022.07
RIF 92770	3.64	4399.95
RIF 116017	4.26	18,004.64
RIF 131214	4.54	34,973.04
RIF 138805	4.44	27,829.03
RIF 143641	5.46	285,648.34
RIF 155841	4.41	25,711.36

**Table 3:** Docking energy of top 10 ligands towards VapC2 (PDB ID 3H87).

Target Protein	Ligands	Docking Energy (kcal mol^−1^)
3H87	Rifampicin	−6.8
	155,841 (Derivative 1)	−8.1
	116,017 (Derivative 2)	−7.9
	138,805 (Derivative 3)	−7.8
	92,770 (Derivative 4)	−7.8
	73,574 (Derivative 5)	−7.8
	25,606 (Derivative 6)	−7.7
	131,214 (Derivative 7)	−7.6
	2074 (Derivative 8)	−7.2
	143,641 (Derivative 9)	−6.9
	21,466 (Derivative 10)	−6.7

**Table 4:** Molecular-docking parameters for the interaction between the target protein and the corresponding inhibitors.

Donor-Acceptor Pairs	Distance (Å)	Type of Interaction	Docking Energy (kcal mol^−1^)	Binding Affinity (M^−1^)
3H87-155841 complex				
LIG: N–GLU57:OE2	5.2186	Electrostatic	−8.1	8.73 × 10^5^
LIG: N–GLU57:OE2	5.5269	Electrostatic		
LIG: N–GLU60:OE1	3.8458	Electrostatic		
LIG:H–GLU57:OE1	2.2104	Conventional Hydrogen Bond		
LIG:C–VAL14	4.6644	Hydrophobic (Alkyl)		
LIG:C–LEU16	3.9970	Hydrophobic (Alkyl)		
A: ARG56–LIG	5.4696	Hydrophobic (Pi-Alkyl)		
LIG–PRO62	4.3944	Hydrophobic (Pi-Alkyl)		
3H87-Rifamycin complex				
ARG56:HH22–LIG: O	2.2590	Conventional Hydrogen Bond	−6.8	9.72 × 10^4^
LIG:C–ILE53	4.9650	Hydrophobic (Alkyl)		
LIG:C–LEU16	4.8045	Hydrophobic (Alkyl)		
LIG:C–PRO62	3.7328	Hydrophobic (Alkyl)		
LIG:C–LEU13	3.6771	Hydrophobic (Alkyl)		
LIG:C–VAL14	3.9681	Hydrophobic (Alkyl)		
LIG–PRO62	4.7323	Hydrophobic (Pi-Alkyl)		

## Data Availability

Not applicable.

## Supplementary Materials

The following supporting information can be downloaded at: https://www.mdpi.com/article/10.3390/molecules28041652/s1, Figure S1: Chemical structures of RIF derivatives 2–10; Figure S2: NMR characterization of derivative 1 (RIF 155841); Figure S3: Mass spectroscopic characterization data of derivative 1 (RIF 155841); Table S1: Binding energy of known *Mtb* drugs with VapC2 protein through blind docking; Table S2: Training dataset with experimental and predicted values; Table S3: Binding energy of 155841_3h87 (VapC2) complex with all poses.
