# Supplementary material for: In Silico Identification of Novel Derivatives of Rifampicin Targeting Ribonuclease VapC2 of M. tuberculosis H37Rv: Rifampicin Derivatives Target VapC2 of Mtb H37Rv
Source: Molecules. 2023 Feb 8;28(4):1652. doi: 10.3390/molecules28041652 (PMC9968056; doi:10.3390/molecules28041652)
Supplement: Supplementary file 1 [file molecules-28-01652-s001.zip › molecules-2032329-supplementary.pdf]

molecules

Supplementary Materials

In Silico Identification of Novel Derivatives of Rifampicin Targeting Ribonuclease VapC2 of *M. tuberculosis* H37Rv: Rifampicin derivatives target VapC2 of *Mtb* H37Rv

Satyamvada Maurya [1], Amita Jain [2], Md Tabish Rehman [3], Ali Hakamy [4], Farkad Bantun [5], Mohamed F. AlAjmi [3], Vineeta Singh [1], Aafreen Zehra [6], Feroz Khan [6], Shafiul Haque [7,8,9] and Bhartendu Nath Mishra [1,*]

* Correspondence: profbnmishra@gmail.com; Tel.: +91-522-2361692; Fax: +91-522-2361631

Table S1. Binding energy of known *Mtb* drugs with VapC2 protein through blind docking.

S.No.	Drugs	Docking energy (kcal mol^{-1})
1	Amikacin	-8.4
2	Amoxicillin	-7.4
3	Bedaquiline	-8.7
4	Cilastatin	-5.9
5	Clavulanate	-6
6	Clofazimine	-7.2
7	Cycloserine	-4.6
8	Ethambutal	-4.6
9	Ethionamide	-5.3
10	Gatifloxacin	-7.1
11	Imipenem	-6.3
12	Isoniazid	-4.9
13	Kanamycin	-7.6
14	Levofloxacin	-6.4
15	Linezolid	-8.1
16	Meropenem	-6.7
17	Moxifloxacin	-6.2
18	Ofloxacin	-6.6
19	Paminosalicylicacid	-5.2
20	Pyrazinamide	-4.4
21	Rifampicin	-8.8
22	Streptomycin	-7.3

Table S2. Training dataset with experimental & predicted values.

Compound No.	Experimental Activity Log10(IC_{50} nM)	Experimental Activity Log10(IC_{50})	Predicted Activity Log10(IC_{50})	Error
C1	11	1.04	1.39	-0.35
C2	5375.16	3.73	3.70	0.04
C3	5914.22	3.77	3.82	-0.05
C4	91	1.96	1.79	0.17
C5	71280	4.85	4.86	-0.01
C6	62340	4.79	3.86	0.94
C7	15510	4.19	3.72	0.47
C8	6250	3.80	3.65	0.14
C9	50259.36	4.70	4.12	0.58
C10	52020	4.72	4.86	-0.14
C11	6918.31	3.84	3.97	-0.13
C12	870.96	2.94	3.74	-0.80
C13	83176.38	4.92	4.00	0.92
C14	3090.3	3.49	3.81	-0.32
C15	3019.975	3.48	4.16	-0.68
C16	10000	4.00	3.77	0.23
C17	6500	3.81	4.74	-0.93
C18	1970	3.29	3.70	-0.40
C19	570	2.76	2.62	0.14
C20	2187.76	3.34	3.10	0.24
C21	26361.34	4.42	4.26	0.16
C22	1174.9	3.07	3.91	-0.84
C23	44350	4.65	4.83	-0.18
C24	61930	4.79	4.75	0.04
C25	1778.28	3.25	3.92	-0.67
C26	4466.84	3.65	3.82	-0.17
C27	72330	4.86	5.14	-0.28
C28	3019.95	3.48	3.90	-0.42
C29	1200	3.08	2.96	0.12
C30	22473.605	4.35	4.71	-0.36
C31	10271.465	4.01	5.15	-1.14
C32	69211.55	4.84	4.38	0.46
C33	30	1.48	2.51	-1.04
C34	3260	3.51	3.81	-0.30
C35	97480	4.99	4.40	0.59
C36	6423.27	3.81	3.89	-0.09
C37	6830	3.83	4.41	-0.58
C38	5011.87	3.70	3.21	0.49
C39	4500	3.65	3.93	-0.28
C40	12000	4.08	4.23	-0.15
C41	33877.21	4.53	4.04	0.49
C42	7109.73	3.85	4.40	-0.55
C43	51353.07	4.71	4.19	0.52
C44	46846.755	4.67	4.31	0.36
C45	22288.605	4.35	4.35	0.00
C46	28056.915	4.45	4.09	0.35

C47	96	1.98	2.06	-0.08
C48	95484.63	4.98	3.81	1.17
C49	26915.35	4.43	3.92	0.51
C50	10495.645	4.02	3.99	0.03
C51	7212.18	3.86	3.93	-0.07
C52	9771.185	3.99	4.08	-0.09
C53	150	2.18	1.87	0.31
C54	12882.5	4.11	4.00	0.11
C55	7000	3.85	3.91	-0.06
C56	6810	3.83	3.81	0.02
C57	68500	4.84	4.88	-0.04
C58	43470.79	4.64	3.76	0.88
C59	21983.81	4.34	4.87	-0.53
C60	147.1428571	2.17	2.55	-0.38
C61	7436.55	3.87	3.70	0.17
C62	43000	4.63	4.97	-0.33
C63	250	2.40	2.40	0.00
C64	1330	3.12	2.88	0.24
C65	320	2.51	2.47	0.04
C66	12644.625	4.10	4.23	-0.13
C67	2640.135	3.42	4.55	-1.13
C68	51	1.71	2.63	-0.92
C69	83130	4.92	4.97	-0.05
C70	21	1.32	1.55	-0.23
C71	16635	4.22	3.92	0.30
C72	1170	3.07	3.40	-0.33
C73	6880	3.84	3.71	0.13
C74	92960	4.97	4.18	0.78
C75	86	1.93	2.71	-0.77
C76	5	0.70	1.71	-1.01
C77	56234.13	4.75	3.93	0.82
C78	8104.155	3.91	4.41	-0.50
C79	17	1.23	1.55	-0.32
C80	73410	4.87	4.30	0.56
C81	19400	4.29	3.59	0.70
C82	13182.57	4.12	3.86	0.26
C83	316.23	2.50	2.77	-0.27
C84	58940	4.77	4.38	0.39
C85	22908.68	4.36	4.11	0.25
C86	4570	3.66	4.21	-0.55
C87	50	1.70	1.72	-0.02
C88	20000	4.30	3.00	1.30
C89	85510	4.93	3.99	0.94
C90	250	2.40	2.40	0.00
C91	26236.34	4.42	3.25	1.17
C92	12500	4.10	3.66	0.44
C93	1513.56	3.18	3.09	0.09
C94	8303.82	3.92	4.40	-0.48
C95	9500	3.98	3.79	0.19
C96	13000	4.11	3.93	0.18
C97	12091.32	4.08	4.38	-0.30

Table S3. Binding energy of 155841_3h87 (VapC2) complex with all poses.

S.No.	Poses	Docking energy (kcal mol^{-1})
1	Pose1	-8.1
2	Pose2	-7.1
3	Pose3	-6.9
4	Pose4	-6.9
5	Pose5	-6.7
6	Pose6	-6.4
7	Pose7	-6.3
8	Pose8	-6.0
9	Pose9	-6.0

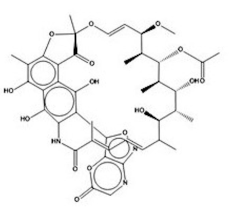

2074

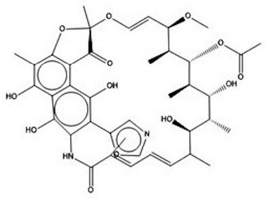

21466

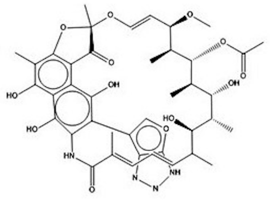

25606

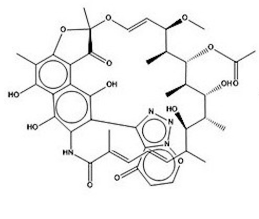

73574

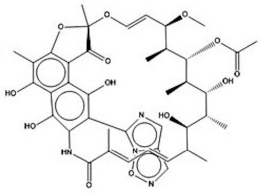

92770

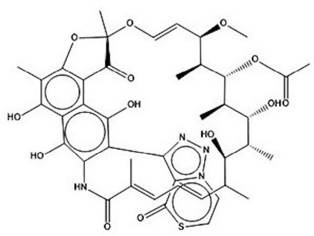

116017

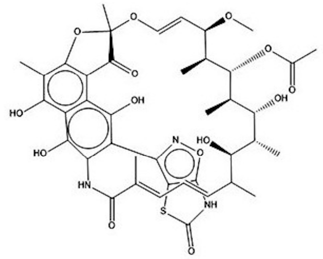

131214

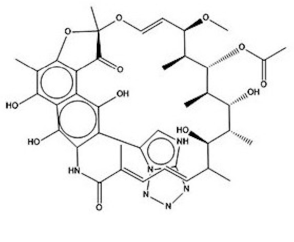

138805

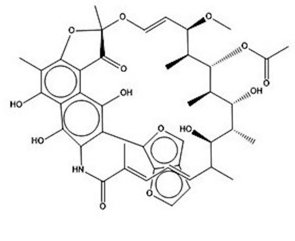

143641

Figure S1. Chemical structures of RIF derivatives 2-10.

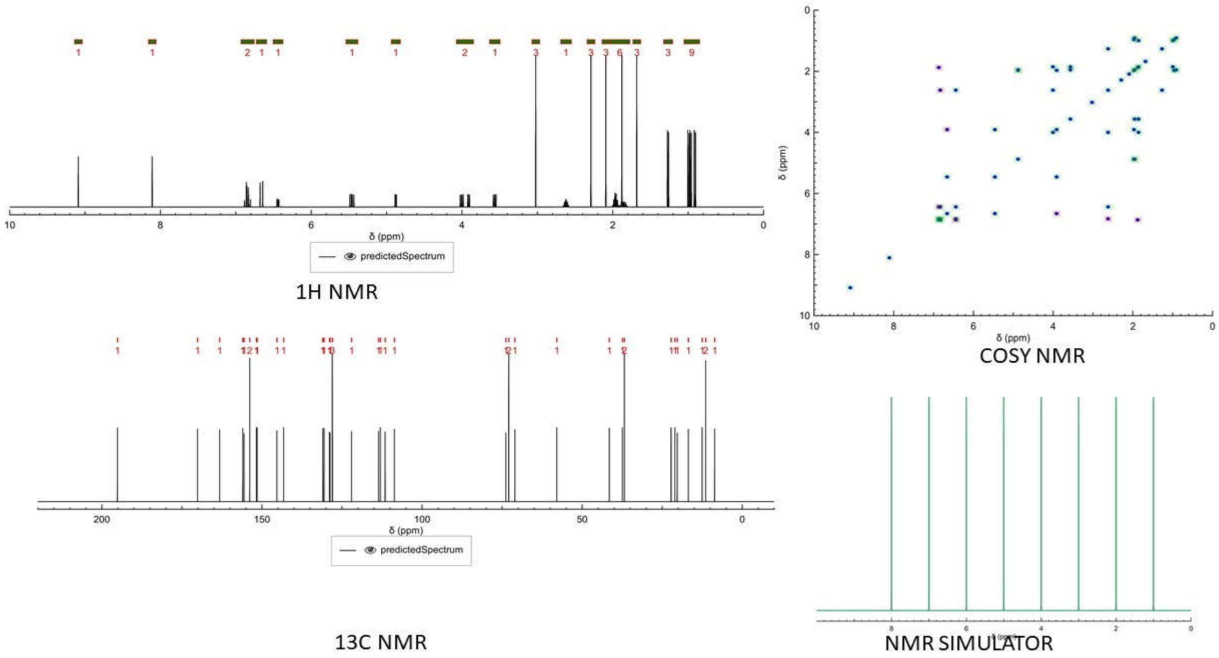

Figure S2. NMR characterization of derivative 1 (RIF 155841).

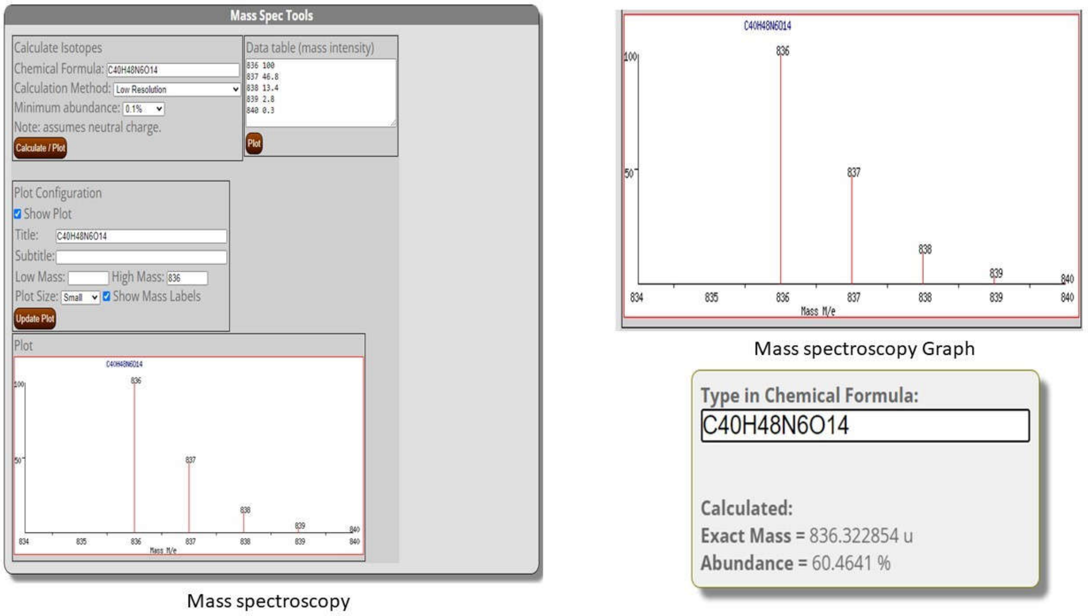

Figure S3. Mass spectroscopic characterization data of derivative 1 (RIF 155841).
